# Genome Sequence of a Salmonella enterica Serotype Senftenberg Strain Lacking *Salmonella* Pathogenicity Island-1 and Isolated in Japan

**DOI:** 10.1128/MRA.00653-19

**Published:** 2019-08-15

**Authors:** Masatomo Morita, Keiji Shimada, Hiroshi Baba, Kei Morofuji, Shunichi Oda, Hidemasa Izumiya, Makoto Ohnishi

**Affiliations:** a Department of Bacteriology I, National Institute of Infectious Diseases, Tokyo, Japan; b Osaka Branch, Japan Food Research Laboratories, Osaka, Japan; c Saito Laboratory, Japan Food Research Laboratories, Osaka, Japan; d Kyushu Branch, Japan Food Research Laboratories, Fukuoka, Japan; University of Maryland School of Medicine

## Abstract

We report here the genome sequence of a Salmonella enterica serotype Senftenberg strain, isolated from feather meal, in which the Salmonella pathogenicity island-1 was deleted.

## ANNOUNCEMENT

Salmonella enterica is a leading cause of bacterial foodborne diseases worldwide (1). Salmonella enterica strain SL180013 was isolated from feather meal as follows: preenrichment was performed with 25 g of the test materials using 250 ml of buffered peptone water (Oxoid Ltd., UK) supplemented with 0.6% polysorbate 20 at 36°C for 18 to 21 h. Selective enrichment was performed using Rappaport-Vassiliadis broth (Oxoid Ltd.) and Hajna’s tetrathionate broth (Eiken Chemical Co. Ltd., Japan) at 42°C for 18 to 24 h. The strain was isolated using deoxycholate-hydrogen sulfide-lactose (DHL) agar (Eiken Chemical Co. Ltd.) and brilliant green agar (Difco, BD Diagnostics, USA). This strain was negative for the *invA* gene, as revealed by PCR (using invA-1 [5′-CGATCTCGATAAAGTCTCTACAG-3′] and invA-2 [5′-ATATACGTTGTACCGTGGCATGTC-3′] as primers), and negative for hydrogen sulfide production, which are common markers for the detection and/or identification of *Salmonella* spp. Despite these results, the strain was identified as Salmonella enterica subsp. enterica by matrix-assisted laser desorption ionization–time of flight mass spectrometry (MALDI-TOF MS; Vitek; bioMérieux, Inc., USA) and serotyped as Senftenberg according to the Kauffmann-White scheme. After culturing in buffered peptone water overnight at 37°C, genomic DNA was extracted using Genomic-tip 100/G (Qiagen, Germany), following the manufacturer’s instructions. A library was prepared using the Nextera XT DNA library preparation kit and processed in a MiSeq platform (Illumina) to obtain paired-end (2 × 300-mer) short reads. Raw sequence reads were filtered using the MiSeq sequence system software (Illumina) to remove low-quality sequences and trimmed to remove adaptor sequences. The genomic DNA was also subjected to sequencing library preparation using the rapid sequencing kit SQK-RAD004 for sequencing with the MinION device (Oxford Nanopore Technologies, UK) (2). The raw data were basecalled using Guppy v2.1.3, and before assembly, adaptors were removed using Porechop v0.2.4 (https://github.com/rrwick/Porechop). In total, 1,441,598 short reads (379,302,334 bp) and 128,804 long reads (560,834,401 bp; median read quality, 8.67) were generated. Default parameters were used for all software programs, unless otherwise specified. A hybrid genome assembly was performed using Unicycler v0.4.4 with the “conservative” mode (3), and three circular contigs were obtained, with sizes of 4,980,580 bp, 4,096 bp, and 2,345 bp. The sequence coverages of the 4,980,580-bp chromosome were 82× for short reads and 107× for long reads. The 4,096-bp plasmid and 2,345-bp plasmid had sequence coverages of 508× and 1,166× for short reads and 1,327× and 1,911× for long reads, respectively. Annotation was performed using the DDBJ Fast Annotation and Submission Tool (DFAST) (4). The overall GC content was 52.3%, and the number of coding sequences was 4,669. Multilocus sequence typing was performed using MLST 2.0 from the assembled genome (5). The strain was typed as sequence type 217 (ST-217). ST-217 belongs to eBG30, which is common to S. enterica serotype Senftenberg (6).

The *Salmonella* pathogenicity island-1 (SPI-1), which includes *invA,* was absent in this strain. The missing region was similar to that of the S. enterica serotype Senftenberg strain N17-509 (7). A nonsense mutation was found at nucleotide position 1621 of *phsA,* encoding thiosulfate reductase, a key enzyme for hydrogen sulfide production (8).

### Data availability.

These genome sequences have been deposited in DDBJ/ENA/GenBank under the accession numbers AP019692, AP019693, and AP019694. The raw sequence data were deposited in the DDBJ Sequence Read Archive under accession number DRR178302.
